# Supplementary material for: Molecular mechanics underlying flat-to-round membrane budding in live secretory cells
Source: Nat Commun. 2022 Jun 27;13:3697. doi: 10.1038/s41467-022-31286-4 (PMC9237132; doi:10.1038/s41467-022-31286-4)
Supplement: Supplementary file 1 — Supplementary Information [file 41467_2022_31286_MOESM1_ESM.pdf]

Supplementary Information

Supplementary information contains Supplementary Figures 1-8.

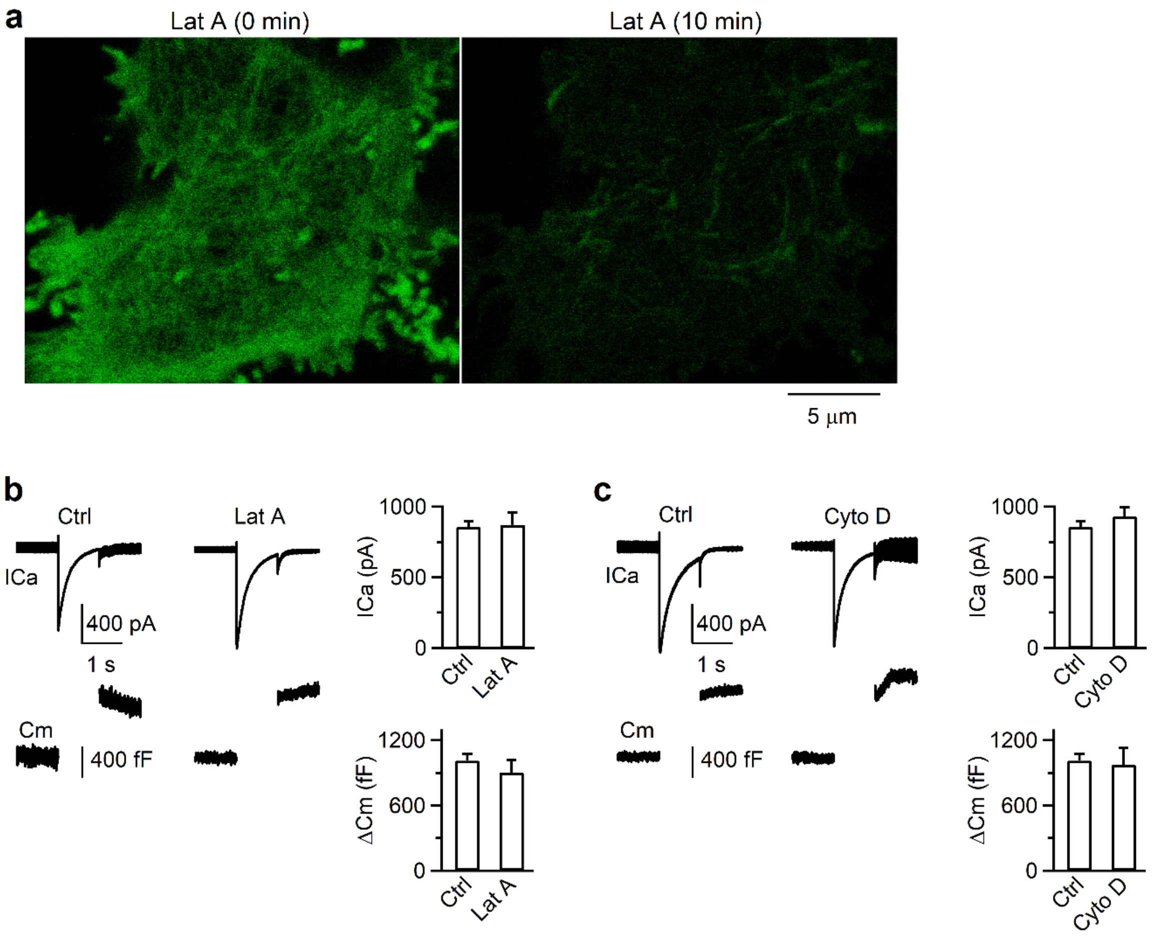

Supplementary Figure 1. Latruculin A or Cytochalasin D discupts F-actin, but does not affect ICa and capacitance jump.

(**a**) F-actin labelled with lifeact-mNeonGreen before (0 min) and 10 min after Latruculin A (Lat A, 3 μM) application in a chromaffin cell (confocal images).

(**b**) Left: sampled ICa (upper) and Cm (lower) induced by $depol_{1s}$ in control.
Middle: sampled ICa (upper) and Cm (lower) induced by $depol_{1s}$ in the presence of Lat A (3 μM).
Right: The amplitude (mean + s.e.m.) of ICa (upper) and ΔCm amplitude (lower) induced by $depol_{1s}$ in control (513 cells from 168 cultures; 336 bovines) and in the presence of Lat A (3 μM, 61 cells from 15 cultures; 30 bovines).

(**c**) Similar arrangements as in **b**, except in control (513 cells from 168 culture; 336 bovines) or in the presence of Cytochalasin D (Cyto D, 4 mM, 57 cells from 12 cultures; 24 bovines). Source data are provided as a Source Data file.

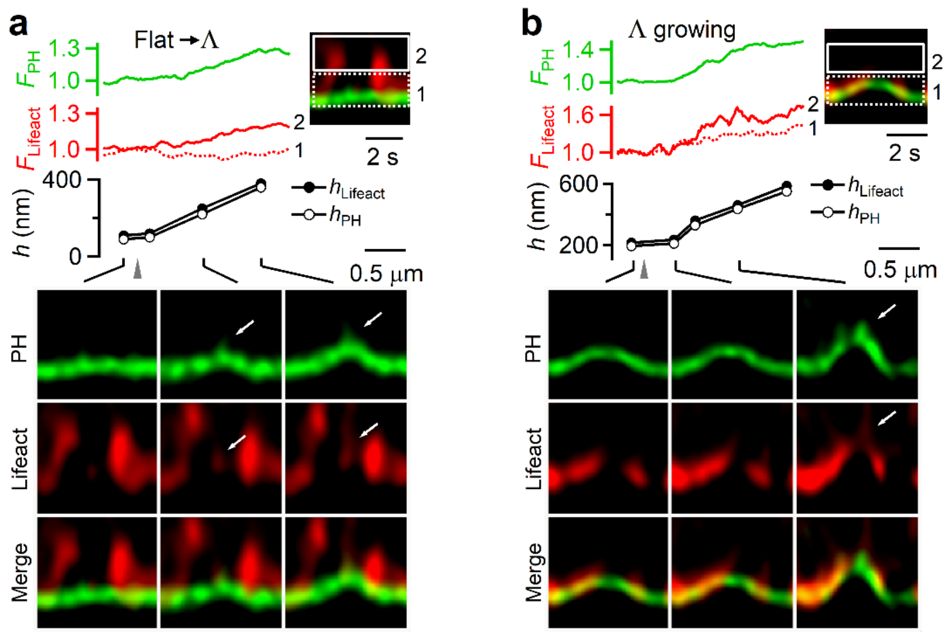

Supplementary Figure 2. F-actin in the act of pulling membrane inward

(**a-b**) F_{PH}, lifeact-mTFP1 fluorescence ($F_{lifeact}$), PH_G-labelled Λ's h_{PH}, the highest position of lifeact-mTFP1-labelled F-actin associated with Λ ($h_{lifeact}$), and sample XZ/Y_{fix} images showing F-actin filament recruitment, attachment at, and movement with the growing Λ's tip during Flat→Λ. $F_{lifeact}$ from regions 1 (near Λ's base) and 2 (above Λ's base, inset) are plotted. spike-like protrusion attached to growing F-actin filaments (arrows). h_{PH} and $h_{lifeact}$ were measured as the height from the PH_G-labelled base membrane. **a** and **b** show two examples; they are the same as Figs 1f and 1g, respectively, except that lifeact fluorescence is plotted with less saturation. Such a plot allowed us to see strong lifeact signals better, but weak signals worse (e.g., lifeact at Λ's tip). Source data are provided as a Source Data file.

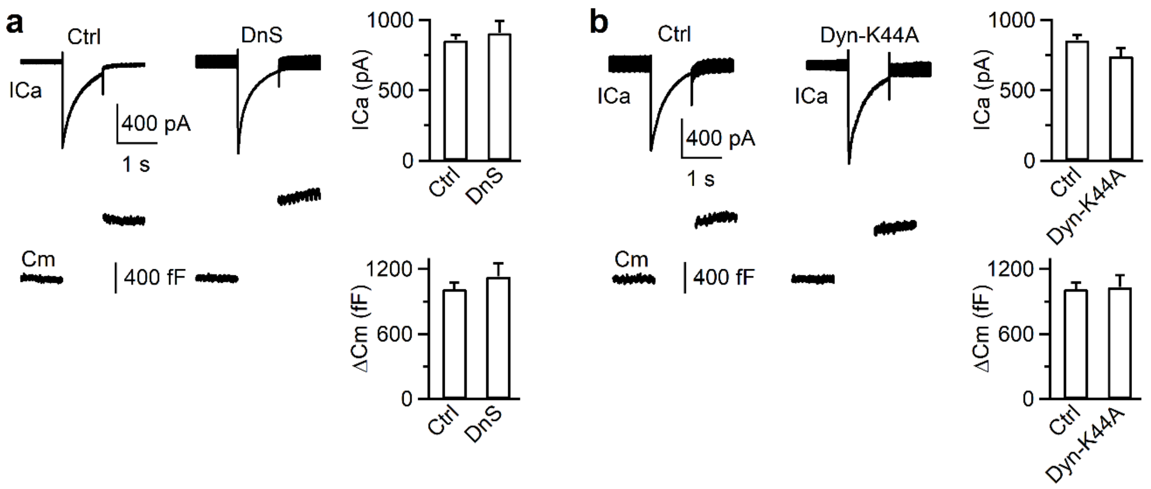

Supplementary Figure 3. Inhibition of dynamin does not affect calcium currents or capacitance jumps.

(**a**) Left: sampled ICa (upper) and Cm (lower) induced by $depol_{1s}$ in control.

Middle: sampled ICa (upper) and Cm (lower) induced by $depol_{1s}$ in the presence of dynasore (80 μM).

Right: The amplitude (mean + s.e.m.) of ICa (upper) and ΔCm amplitude (lower) induced by $depol_{1s}$ in control (513 cells from 168 cultures; 336 bovines) and in the presence of dynasore (DnS, 80 μM, 64 cells from 13 cultures; 26 bovines).

(**b**) Similar arrangements as in panel a, except in different conditions: without (513 cells from 168 cultures; 336 bovines; Ctrl) or with overexpression of dynamin 1-K44A (Dyn-K44A, 106 cells from 21 cultures; 42 bovines). Source data are provided as a Source Data file.

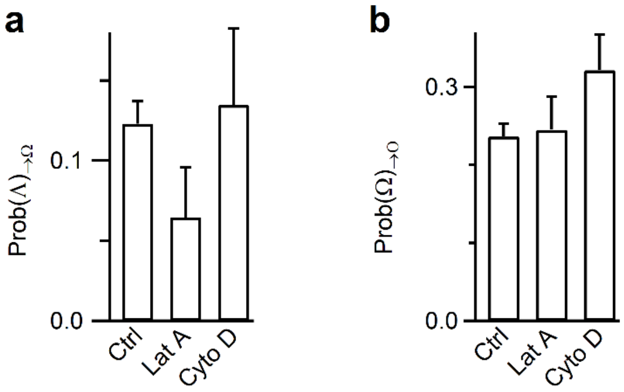

Supplementary Figure 4. Inhibition of F-actin does not significantly affect Λ→Ω or Ω→O transition.

(**a**) Prob(Λ)$_{\rightarrow\Omega}$ in control (Ctrl, 513 cells from 168 cultures; 336 bovines), latrunculin A (Lat A, 61 cells from 15 cultures; 30 bovines, $p = 0.183$, two-tailed unpaired t test, compared to Ctrl), or Cytochalasin D (Cyto D, 57 cells from 12 cultures; 24 bovines, $p = 0.903$, two-tailed unpaired t test, compared to Ctrl).

(**b**) Prob(Ω)$_{\rightarrow O}$ in control (Ctrl, 513 cells from 168 cultures; 336 bovines), latrunculin A (Lat A, 61 cells from 15 cultures; 30 bovines, $p = 0.305$, two-tailed unpaired t test, compared to Ctrl), or Cytochalasin D (Cyto D, 57 cells from 12 cultures; 24 bovines, $p = 0.068$, two-tailed unpaired t test, compared to Ctrl). Source data are provided as a Source Data file.

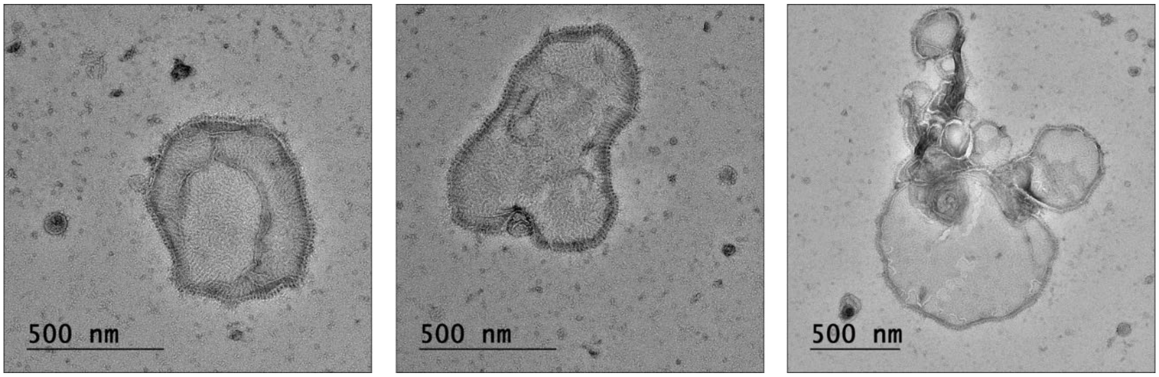

Supplementary Figure 5. Dynamin decoration of large liposomes

Sampled negative stain images of ΔPRD-dynamin 1 decorated around large DOPS vesicles after 30 sec of incubation with the lipid. Similar results were obtained from 104 micrographs in 3 independent experiments.

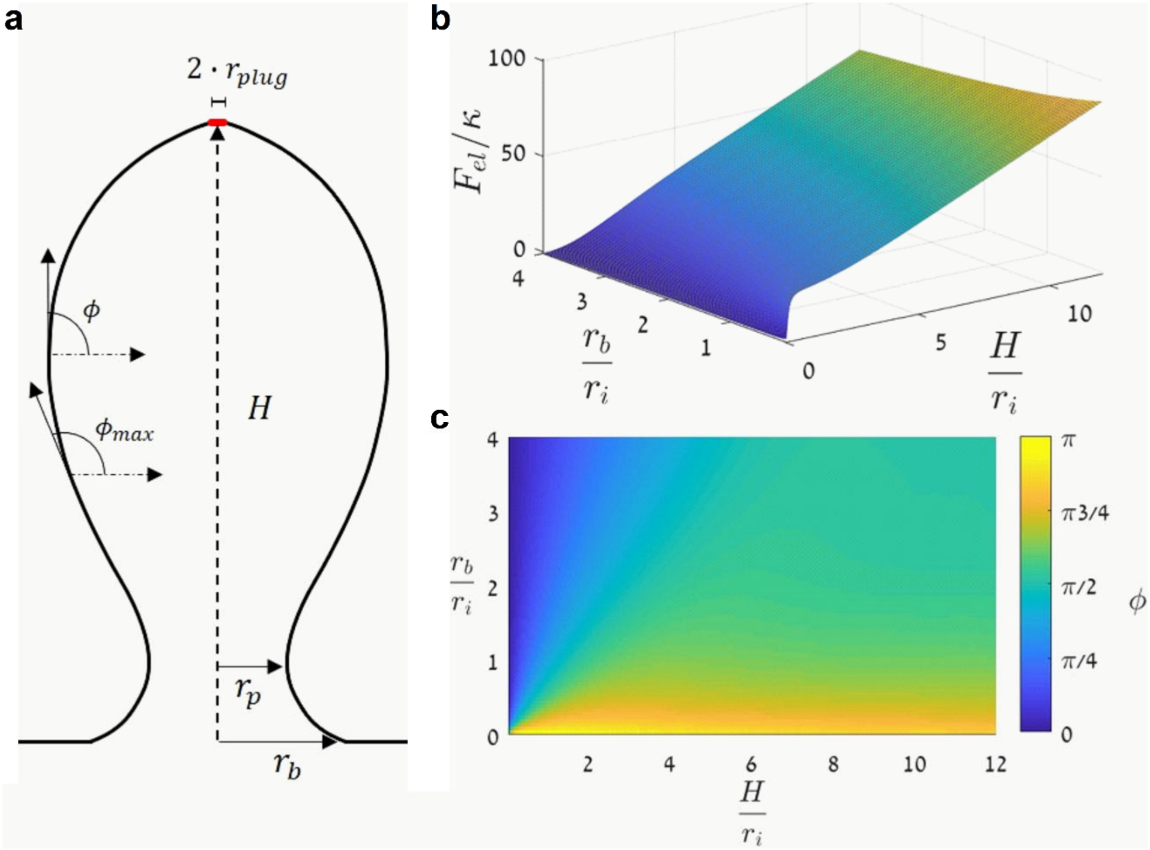

Supplementary Figure 6. Model and parameter definitions.

(**a**) Parameters of the membrane shape: H is the height, r_b is the base edge radius, r_p is the pore radius, ϕ and ϕ_{max} are the tangent angle of the membrane profile and is its maximal value, r_{plug} is the radius of a circular membrane area to which the pulling force, f_{pull}, is applied.

(**b**) The energy landscape representing the system elastic energy normalized by the bending rigidity, $\frac{F_{el}}{\kappa}$, as a function of the normalized geometrical parameters, $\frac{H}{r_i}$ and $\frac{r_b}{ri}$.

(**c**) Shape diagram representing in terms of the parameters $\frac{H}{r_i}$ and $\frac{r_b}{r_i}$, where $r_i = \sqrt{\frac{\kappa}{2\gamma_0}}$ is the internal length scale, κ is the membrane bending modulus, γ_0 is the membrane tension. The diagram represents the maximal tangent angle, ϕ_{max}, which is colored coded and spans the range from low angles for flat shapes, through values close to $\frac{\pi}{2}$ for tubular shapes, and up to angles higher than $\frac{\pi}{2}$ for Ω shapes.

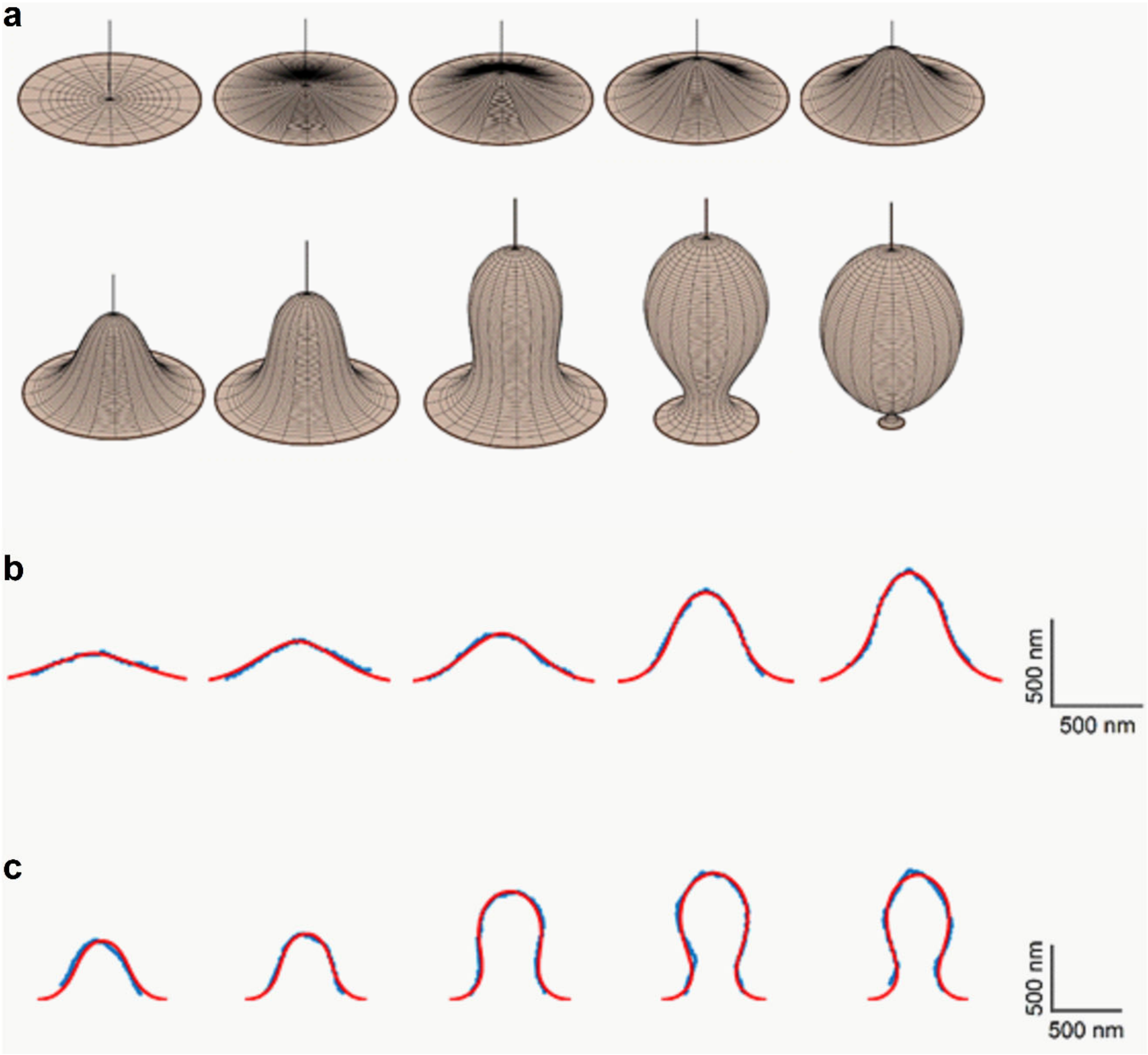

Supplementary Figure 7. Evolution of bud shape.

(**a**) Three-dimensional view of the computed membrane configurations representing the typical sequence of events: upper panel, Λ shapes with increasing height; lower panel, transformation of Λ-to Ω- shapes.

(**b-c**) Fitting of the computed shape profiles to the time sequence of shapes observed experimentally. The experimentally observed shape profiles were processed into pixel series shown by blue squares. The fitted model profiles are presented by red curves. The overlap between the fitted profiles and the original experimental images are presented in the main text (Figs. 7e and 7f, respectively).

b, Flat→Λ transition, see also Movie S7 for the computed versus the experimentally observed shape changes.

c, Λ→Ω transition, see also Movie S8 for the computed versus the experimentally observed shape changes. Source data are provided as a Source Data file.

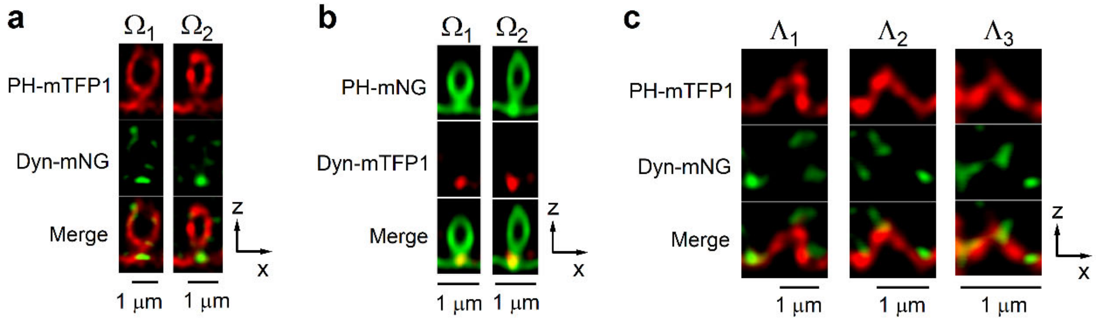

Supplementary Figure 8. Dynamin 2-mNG association with PH-mTFP1-labelled Ω- and Λ-profiles

(a) XZ-plane STED images of PH-mTFP1 and dynamin 2-mNG (Dyn-mNG) showing dynamin at the pore region of Ω-profiles (Ω_1 and Ω_2 are different Ω-profiles). Similar results were observed from 10 events (4 cultures, 8 bovines).

(b) XZ-plane STED images of PH-mNG and dynamin 2-mTFP1 (Dyn-mTFP1) showing dynamin at the pore region of Ω-profiles (Ω_1 and Ω_2 are different Ω-profiles). Similar results were observed from 15 events (6 cultures, 12 bovines).

(c) XZ-plane STED images of PH-mTFP1 and dynamin 2-mNG (Dyn-mNG) showing dynamin association with Λ-profiles (Λ_1, Λ_2 and Λ_3 are different Λ-profiles). Similar results were observed from 9 events (4 cultures, 8 bovines).
