# Supplementary material for: Molecular mechanics underlying flat-to-round membrane budding in live secretory cells
Source: Nat Commun. 2022 Jun 27;13:3697. doi: 10.1038/s41467-022-31286-4 (PMC9237132; doi:10.1038/s41467-022-31286-4)
Supplement: Supplementary file 3 — Description of Additional Supplementary Files [file 41467_2022_31286_MOESM3_ESM.pdf]

Supplementary Video Legends

Supplementary Movie 1. Actin filament in the act of pulling membrane inward to form Λ.

Horizontal length of the image frame is 1.7 μm. The event is the same as the one shown in Figure 1f. Green: PH_G-labelled plasma membrane; red: Lifeact-labelled F-actin. The video is played at real-time.

Supplementary Movie 2. Dynamin in the act of constricting Λ's base and Ω's pore

Horizontal length of the image frame is 1.7 μm. The event is the same as the one shown in Figure 4c. Green: PH_G-labelled plasma membrane; red: dynamin 1-mTFP1 puncta. The video is played 2 times as fast as the real-time.

Supplementary Movie 3. Cryo-electron tomogram of dynamin 1 with DOPS vesicles

3-dimensional views of dynamin 1 assembled around the DOPS vesicle. Data are the same as those shown in Figure 5d.

Supplementary Movie 4. CryoET segmentation of dynamin 1 around DOPS vesicle

Dynamin 1 (yellow) forms helices around the DOPS vesicle (grey) with a diameter of ~166 nm. Data are the same as those shown for Figure 5e, upper row.

Supplementary Movie 5. Cryo-ET segmentation of dynamin 1 around DOPS vesicle

Dynamin 1 (yellow) forms helices around the DOPS vesicles (grey) with a diameter of ~85 nm. Data are the same as those shown for Figure 5e, lower row.

Supplementary Movie 6. Simulated shape evolution for the Flat$\rightarrow\Lambda\rightarrow\Omega$ transition

Evolution of simulated 3-D shapes for Flat$\rightarrow\Lambda$ (Figure 7c) and $\Lambda\rightarrow\Omega$ (Figure 7d) transitions combined together, viewed from a different angle. The values of the pulling force, f_{pull}, and the base radius, r_b, are shown for each frame. All lengths are scaled by the intrinsic length, r_i.

Increasing the pulling force, f_{pull}, while constraining the base to a constant value, $r_b = 2.5 \cdot r_i$, results in Flat to Λ transition, which corresponds to the curve shown in (Figure 7c). The shape transformation continues smoothly from Λ to Ω–shape by the base constriction upon conservation of the pulling force ($f_{pull} \cong 7.2 - 7.3\ f_i$) and membrane area, which corresponds to the curve shown in (Figure 7d).

Supplementary Movie 7. Flat→Λ transition: the observed versus fitted simulation profiles

Left: real-time video of the experimentally observed membrane profile evolution (scale bar: 500 nm).

Right: corresponding sequence of model-derived profiles obtained by fitting of the model parameters (see theoretical mathematical modeling). The fitted pulling force, f_{pull}, is labelled in every video frame.

Supplementary Movie 8. Λ→Ω transition: the observed versus fitted simulation profiles

Left: real-time video of the experimentally observed membrane profile evolution (scale bar: 500 nm).

Right: corresponding sequence of model-derived profiles obtained by fitting of the model parameters (see theoretical mathematical modeling). The fitted base diameter, r_b, is labelled in every video frame.
