# Supplementary material for: Molecular mechanics underlying flat-to-round membrane budding in live secretory cells
Source: Nat Commun. 2022 Jun 27;13:3697. doi: 10.1038/s41467-022-31286-4 (PMC9237132; doi:10.1038/s41467-022-31286-4)
Supplement: Supplementary file 12 — Reporting summary [file 41467_2022_31286_MOESM12_ESM.pdf]

nature portfolio

Corresponding author(s): Jenny Hinshaw, Michael Kozlov, Ling-Gang Wu

Last updated by author(s): May/13/22

Reporting Summary

Nature Portfolio wishes to improve the reproducibility of the work that we publish. This form provides structure for consistency and transparency in reporting. For further information on Nature Portfolio policies, see our Editorial Policies and the Editorial Policy Checklist.

Statistics

For all statistical analyses, confirm that the following items are present in the figure legend, table legend, main text, or Methods section.

n/a	Confirmed	
☐	☒	The exact sample size (n) for each experimental group/condition, given as a discrete number and unit of measurement
☐	☒	A statement on whether measurements were taken from distinct samples or whether the same sample was measured repeatedly
☐	☒	The statistical test(s) used AND whether they are one- or two-sided<br>*Only common tests should be described solely by name; describe more complex techniques in the Methods section.*
☐	☒	A description of all covariates tested
☐	☒	A description of any assumptions or corrections, such as tests of normality and adjustment for multiple comparisons
☐	☒	A full description of the statistical parameters including central tendency (e.g. means) or other basic estimates (e.g. regression coefficient) AND variation (e.g. standard deviation) or associated estimates of uncertainty (e.g. confidence intervals)
☐	☒	For null hypothesis testing, the test statistic (e.g. F, t, r) with confidence intervals, effect sizes, degrees of freedom and P value noted<br>*Give P values as exact values whenever suitable.*
☒	☐	For Bayesian analysis, information on the choice of priors and Markov chain Monte Carlo settings
☒	☐	For hierarchical and complex designs, identification of the appropriate level for tests and full reporting of outcomes
☒	☐	Estimates of effect sizes (e.g. Cohen's d, Pearson's r), indicating how they were calculated

Our web collection on statistics for biologists contains articles on many of the points above.

Software and code

Policy information about availability of computer code

Data collection	Leica LAS X was used to acquire images. Pulse 8.74 was used to acquire patch-clamp electrophysiology data.
Data analysis	Image J, Leica LAS X, and Huygens software were used for data analysis for images and patch-clamp electrophysiological data, respectively. MATLAB R2019a was used for all calculations, image processing, and fitting. The main computational codes used for the simulations and fitting procedure in this paper are available at https://github.com/benzucker-tau/Endocytosis.

For manuscripts utilizing custom algorithms or software that are central to the research but not yet described in published literature, software must be made available to editors and reviewers. We strongly encourage code deposition in a community repository (e.g. GitHub). See the Nature Portfolio guidelines for submitting code & software for further information.

Data

Policy information about availability of data

All manuscripts must include a data availability statement. This statement should provide the following information, where applicable:

- Accession codes, unique identifiers, or web links for publicly available datasets
- A description of any restrictions on data availability
- For clinical datasets or third party data, please ensure that the statement adheres to our policy

The data that support the findings of this study are available from corresponding authors upon reasonable request.

Field-specific reporting

Please select the one below that is the best fit for your research. If you are not sure, read the appropriate sections before making your selection.

- [x] Life sciences
- [ ] Behavioural & social sciences
- [ ] Ecological, evolutionary & environmental sciences

For a reference copy of the document with all sections, see nature.com/documents/nr-reporting-summary-flat.pdf

Life sciences study design

All studies must disclose on these points even when the disclosure is negative.

Sample size

In Chromaffin cells, the sample size in this study is from at least 11 cells, which are taken from 3 or more independent cultures, number of cells for each condition are indicated in the results and figure legends. This allowed us to perform statistical analysis as described in the figure legends. The sample size is also based on our previous experience with the same cells and similar techniques to obtain consistent results. The actual sample size are presented with each result in the main text of the manuscript. In hippocampal neurons, the sample size is from 40-100 synaptic profiles from 3-8 independent cultures. In Cryo EM experiemts, the statistics were performed using unpaired t test with Welch's correction where n represents diameter (nm) of every dynamin decorated tube and vesicle. The sample size was decided based on more than ten years of experience in the study of this field, as referenced below.

1. Shin, W. et al. Preformed Omega-profile closure and kiss-and-run mediate endocytosis and diverse endocytic modes in neuroendocrine chromaffin cells. Neuron 109, 3119-3134 e3115 (2021).
2. Shin, W. et al. Visualization of Membrane Pore in Live Cells Reveals a Dynamic-Pore Theory Governing Fusion and Endocytosis. Cell 173, 934-945 (2018).
3. Zhao, W.D. et al. Hemi-fused structure mediates and controls fusion and fission in live cells. Nature 534, 548-552 (2016).
4. Wen, P.J. et al. Actin dynamics provides membrane tension to merge fusing vesicles into the plasma membrane. Nat. Commun 7, 12604 (2016).
5. Chiang, H.C. et al. Post-fusion structural changes and their roles in exocytosis and endocytosis of dense-core vesicles. Nat. Commun 5, 3356 (2014).
6. Wu, X.S. et al. Presynaptic Kv3 channels are required for fast and slow endocytosis of synaptic vesicles. Neuron 109, 938-946 e935 (2021).
7. Wu, X.S. et al. Actin Is Crucial for All Kinetically Distinguishable Forms of Endocytosis at Synapses. Neuron 92, 1020-1035 (2016).
8. Zhang, Z. et al. The SNARE proteins SNAP25 and synaptobrevin are involved in endocytosis at hippocampal synapses. J. Neurosci 33, 9169-9175 (2013).
9. Sun, T. et al. The role of calcium/calmodulin-activated calcineurin in rapid and slow endocytosis at central synapses. J Neurosci 30, 11838-11847 (2010).

Data exclusions

There are no data exclusions. We have included all images and traces from STED, patch-clamp electrophysiology and Cryo EM.

Replication

STED imaging and patch-clamp electrophysiology experiments were repeated in at least 11 cells (up to 513 cells) for each group of data, which were taken from 3 or more independent cultures. For each group of data, the cell number, culture number, and animal number are specified in the Results and figure legends. Furthermore, our findings were verified by 1-3 people from 3 or more independent experiments in the lab, and all attempts at replication were successful and confirmed with minor statistical differences which are not significant to overturn the our statistical results. For the Cryo EM experiments, the reproducibility of the data of every condition was checked by performing experiments on 3 different days and freezing 4 or more grids of each condition. We repeated each experiment 3 times and 104 micrographs for each condition.

Randomization

All experimental findings were from completely randomized allocated samples.

Blinding

The investigators did not have expected outcomes before performing experiments and analysis. Blinding was not used for data collection or analysis. Bovine selection was blinded – it was selected by workers in the abattoir without our participation.

Reporting for specific materials, systems and methods

We require information from authors about some types of materials, experimental systems and methods used in many studies. Here, indicate whether each material, system or method listed is relevant to your study. If you are not sure if a list item applies to your research, read the appropriate section before selecting a response.

Materials & experimental systems

n/a	Involved in the study	
☒	☐	Antibodies
☐	☒	Eukaryotic cell lines
☒	☐	Palaeontology and archaeology
☐	☒	Animals and other organisms
☒	☐	Human research participants
☒	☐	Clinical data
☒	☐	Dual use research of concern

Methods

n/a	Involved in the study	
☒	☐	ChIP-seq
☒	☐	Flow cytometry
☒	☐	MRI-based neuroimaging

Eukaryotic cell lines

Policy information about cell lines

Cell line source(s)	Sf9 insect cells (ThermoFisher Scientific, Cat #: 11496015)
Authentication	The cell line has not been authenticated
Mycoplasma contamination	Cell line was not tested for mycoplasma contamination but no indication of contamination was observed.
Commonly misidentified lines (See ICLAC register)	No commonly misidentified cell lines were used

Animals and other organisms

Policy information about studies involving animals; ARRIVE guidelines recommended for reporting animal research

Laboratory animals	Wild-type (C57BL/6J) and ActbLoxP/LoxP mice of either sex were used
Wild animals	We acquired adrenal glands from 21-27 month old bovine of either sex from a local abattoir (J. W. Treuth & Sons Inc., 328 Oella Ave, Catonsville, MD 21228; web site: https://www.jwtreuth.com). No animal use protocol was required.
Field-collected samples	No field collected samples were used in the study.
Ethics oversight	NIH Animal Care and Use Committee.

Note that full information on the approval of the study protocol must also be provided in the manuscript.
